# Application of the adenosine triphosphate sensitivity assay in infantile vascular anomalies

**DOI:** 10.1186/s12887-020-1974-9

**Published:** 2020-02-19

**Authors:** Li Li, Bin Yang, Li Wei, Bin Zhang, Xiao-feng Han, Zi-gang Xu, Lin Ma

**Affiliations:** 1 Department of Dermatology, Beijing Children’ s Hospital, Capital Medical University, National Center for Children’ s Health, Beijing, 100045 China; 2 Department of Dermatovenereology, Maanshan people’ s hospital, Maanshan, 243000 China

**Keywords:** Vascular anomalies, Infantile hemangiomas, Capillary malformations, Adenosine triphosphate, Sensitivity assay, In vitro

## Abstract

**Background:**

The term vascular anomalies include various vascular tumors and vascular malformations, among them infantile hemangiomas and capillary malformations are the most well-known associated diseases in early ages. Multiple drugs have been introduced for intervention, but susceptibility test in vitro were scarcely reported.

**Objective:**

To evaluate the inhibition effect of different drugs by adenosine triphosphate sensitivity assay in vitro before the treatment of infantile hemangiomas and capillary malformations.

**Methods:**

Specimens were selected from 5 cases of infantile hemangiomas and 11 cases of capillary malformations. Propranolol, rapamycin, sildenafil and itraconazole were tested for their growth inhibition effect by using the adenosine triphosphate sensitivity assay.

**Results:**

Propranolol demonstrated inhibitory effects on infantile hemangiomas cells. Rapamycin and itraconazole both showed inhibitory effects on infantile hemangiomas cells and capillary malformations cells. Sildenafil has no growth inhibitory effect on infantile hemangiomas cells or capillary malformations cells.

**Conclusion:**

Adenosine triphosphate sensitivity assay is a sensitive and useful testing method before the management of vascular anomalies, and individualized medication suggestions for the choice of therapeutic drugs were offered based on the testing result and together with a comprehensive evaluation of each infant.

## Background

Vascular anomalous entities used to be called in an ambiguous term as “hemangioma” to designate numerous quite different disorders especially in infancy [1]. However, in recent decades, the International Society for the Study of Vascular Anomalies (ISSVA) developed a classification system and these anomalies are divided into vascular tumors and vascular malformations based on their biology and genetics [2].

Infantile hemangiomas are the most common vascular anomalies in infants [3], and it is also the most common benign neoplasm in infants. Its incidence rate is about 5–12%, but in premature or low birth weight infants whose weight were less than 1000 g the incidence rate could reach as high as 22–30% [4]. Besides, vascular malformations are also frequently occurred in infants, of which capillary malformations (naevus flammeus, port wine stains) are the most common entities [5].

There are multiple available treatment prescriptions or approaches for vascular anomalies, including drug therapy (such as glucocorticoids, antineoplastic chemotherapeutic agents, interferon, etc.), physical therapy (such as laser [6], freezing, radiotherapy, radionuclide, radioisotope, and shallow X-ray radiation), surgical intervention, electrochemical therapy, and so on [7]. However, physical therapy and surgical intervention sometimes appeared to be harmful, so the optional effective methods that can be used in infant period, or especially in neonatal period, are relative lacking. In 2008, propranolol was serendipitously discovered to cause regression of proliferating hemangiomas in newborns receiving treatment for other disease [8]. Since then, numerous studies have demonstrated the success of propranolol for shrinking hemangiomas through various forms [9]. Although the specific mechanism has not yet been elucidated, propranolol is more effective than other medical intervention of infantile hemangioma with fewer adverse reactions, and so propranolol gradually replaces those therapies with severe injury and high incidence of complications, and gradually becomes the first-line clinical therapy for infantile hemangioma [10]. In addition, some other beta-blockers (atenolol [11], timolol [12], etc.), itraconazole [13], rapamycin (sirolimus) [14] and sildenafil [15, 16] have been reported to be effective in treating certain vascular anomalies.

Although the above drugs have already been used in clinical treatment of vascular anomalies, they are still in the stage of empirical drug use, lacking theoretical guidance for drug selection and efficacy evaluation. Therefore, referring to and imitating the drug sensitivity test in vitro of antineoplastic drugs [17], we carried out a similar in vitro drug sensitivity test on infantile vascular anomalies lesion tissues before initiating therapy. In laboratory environment, the inhibitory effects on the lesion tissues of the four drugs that were commonly used in treating infantile vascular anomalies were observed and calculated. By comparing the inhibition rates of different drugs on individual tissue cells, the drug evaluation report for specific infant was proposed.

This paper is only the laboratory research report before clinical medication, and the follow-up clinical efficacy observation is still in progress.

## Methods

This study was approved by the institutional review board of the Capital Medical University for medical sciences. All procedures were carried out in full compliance with the requirements of the Code of Ethics of the World Medical Association.

Adenosine triphosphate sensitivity assay in vitro were performed to evaluate the inhibition effect of different drugs on severe infantile hemangiomas or capillary malformations.

### Patients and specimens

Infants aged less than 3 months with severe vascular anomalies which needed to be intervened were randomly enrolled when they were first visit to Outpatient Department, with the informed consent of their parents (inclusion criteria: severe infantile hemangiomas or capillary malformations which growing rapidly, with large sizes, or caused serious deformity or dysfunction, exclusion criteria: vascular anomalies with life-threatening complication that required surgical operation). All cases were diagnosed by clinical and color Doppler ultrasonography. Finally, there are 5 cases of severe infantile hemangiomas, 2 males and 3 females, with an average age of 1.5 months, and 11 cases of severe capillary malformations, 5 males and 6 females, with an average age of 2 months, were included. Written informed consent on participation in drug sensitivity assay in vitro was obtained from both parents in all cases.

The typical vascular anomalies lesions were selected as sampling sites, and lesions with eczema, scale, erosion, ulcer, crust, necrosis or liquefaction occurred or around were excluded. After a minor excision surgery by trephine, the tissues specimens were immersed in a particular sterile vial containing culture liquid and sent to laboratory within 1 h.

### Test drugs

Four drugs that most frequently used in treating infantile vascular anomalies namely propranolol, rapamycin (sirolimus), sildenafil and itraconazole were tested for their sensitivity. The corresponding peak plasma concentration (PPC, Cmax) is calculated and determined based on the pharmacokinetic index, drug instructions or manuals and available literature according to their dosage in pediatrics [18, 19]. As for ease to operate and prepare the reagents, all of the data were taken as integers for convenience. The peak plasma concentration in this study were setting as: propranolol 200 ng/ml, rapamycin 20 ng/ml, sildenafil 200 ng/ml, itraconazole 500 ng/ml. Six testing concentrations were set up to simulate the drug metabolic concentration in plasma, which were 400, 200, 100, 50, 25 and 12.5% of the peak plasma concentration. Each concentration was set up three parallel holes in order to take an average number. Meanwhile, drug-free control (M0, only culture medium and lesions tissues, without any drug) and maximum inhibitory control (MI, only culture medium) were also set up.

This study is only an in vitro laboratory research currently, but according to the previous experimental design, it is planned to be widely used in practice in the future for clinical medication instruction. Therefore, parents are fully encouraged to participated and involved in the formulation of treatment plans and select experimental drugs. The currently available pharmacological mechanism and the administrations dose and pattern of each drug are fully communicated with parents. As fear of or concerned about the adverse reactions of itraconazole, in this study, among 5 cases of infantile hemangioma, three parents chose propranolol, rapamycin, sildenafil and itraconazole as testing drug, but the other two parents only chose propranolol, rapamycin and sildenafil. Among 11 cases of capillary malformations, seven parents chose propranolol, rapamycin, sildenafil and itraconazole and the other four parents only chose propranolol, rapamycin and sildenafil. We fully respect parents’ choices and inclination.

### Drug susceptibility assays

This study was carried out by using the adenosine triphosphate-tumor chemo-sensitivity assay to assess the sensitivity of vascular anomalies cells to a range of therapeutic agents. A self-made kit, containing sterile compound digestive enzymes, erythrocyte scavenger, serum-free culture medium, adenosine triphosphate inhibitor reagent, adenosine triphosphate extract reagent, luciferase-fluorescein reagent, etc. is needed during related subsequent laboratory operation.

The vascular anomalies tissues specimens were dissected and dissociated adequately, and removal of connective tissue, fibers, fat and red blood cells on the surface of specimens. After soaking, digestion and incubation, the suspension of tissues and cells were prepared like a light and fluffy paste or mush. After equivalent culture medium and different concentration of the four testing drugs were adding to the culture microplate, the suspension of vascular anomalies tissues and cells were cultured into the medium.

The suspensions were incubated at 37 °C for 3 days. After that, adenosine triphosphate extract reagents were used to extract adenosine triphosphate from the suspensions, and then, the luciferase-fluorescein reagents were added for fluorescence assay.

The fluorescence intensity of these specimens at 560 nm wavelength was measured by fluorescence scanner and illuminometer, which represented and symbolized the levels of adenosine triphosphate and the numbers of viable cell remaining at present. Data were recorded and assessed, and the mean values of three parallel holes were calculated. Combined with MI and M0 and conducted a comprehensive analysis (M0, without any drug, only culture medium and lesions tissues, its testing results indicated the situation of cell culture and natural apoptosis), the actual inhibition rate of the six concentrations of each drug in every specimens was determined and identified. Then, the average inhibition rates of each drug in two groups, infantile hemangiomas group and capillary malformations group, were calculated respectively. Intuitively, inhibition curves could plot and draw when necessary.

### Data analysis

In traditional adenosine triphosphate-tumor chemo-sensitivity assay, researchers isolate cancer cells from patient neoplasm, culture them and expose them to an array of drugs to assess response. By reference to the M0, these assays typically assess response using cell survival/death rate and a series of related parameters: including IC50, IC90, TGI, SI, AUC, etc. [20]. However, considering that all testing drugs in this study are non-cytotoxic and have no cytocidal activity, it is difficult to achieve 50% (IC50) or even 90% (IC90) inhibition rate as anti-cancer drugs. Simultaneously, sensitivity index (SI) > 250 is also hard to reach [21], and the area under curve (AUC) is very small. Accordingly, we set that when an inhibition rate is up to 20% at a certain concentration could be considered effective, and adopt a total growth inhibition rate (TGI, summation of the percentage of growth inhibition at every testing concentration) to compare the differences between various drugs. Besides, the general situation of the application of each drug in the two anomalies groups, infantile hemangiomas group and capillary malformations group, was analyzed and discussed. As these sample sizes were too small, neither statistical hypothesis test nor difference analysis was done.

## Results

**Fig. 1:**
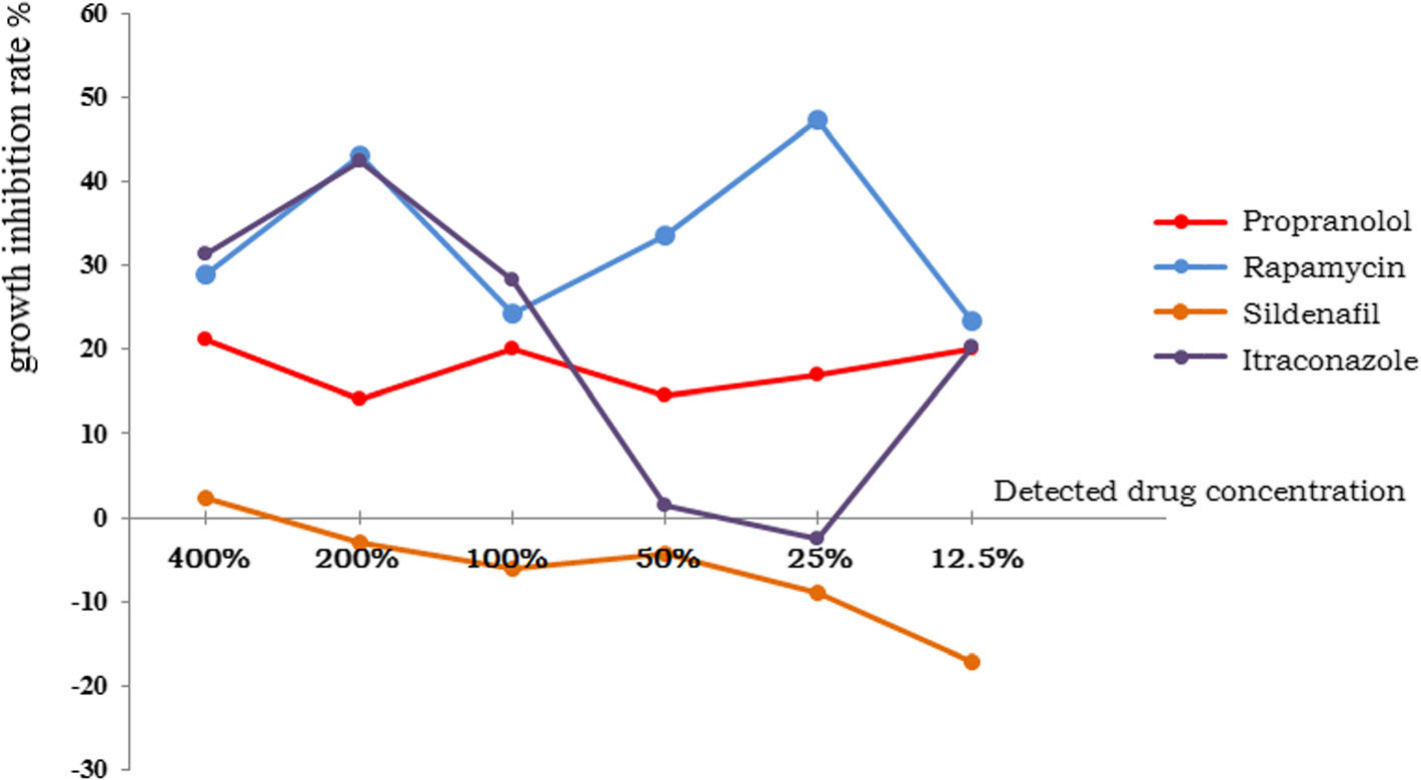
The average inhibition curves of infantile hemangiomas group

**Fig. 2:**
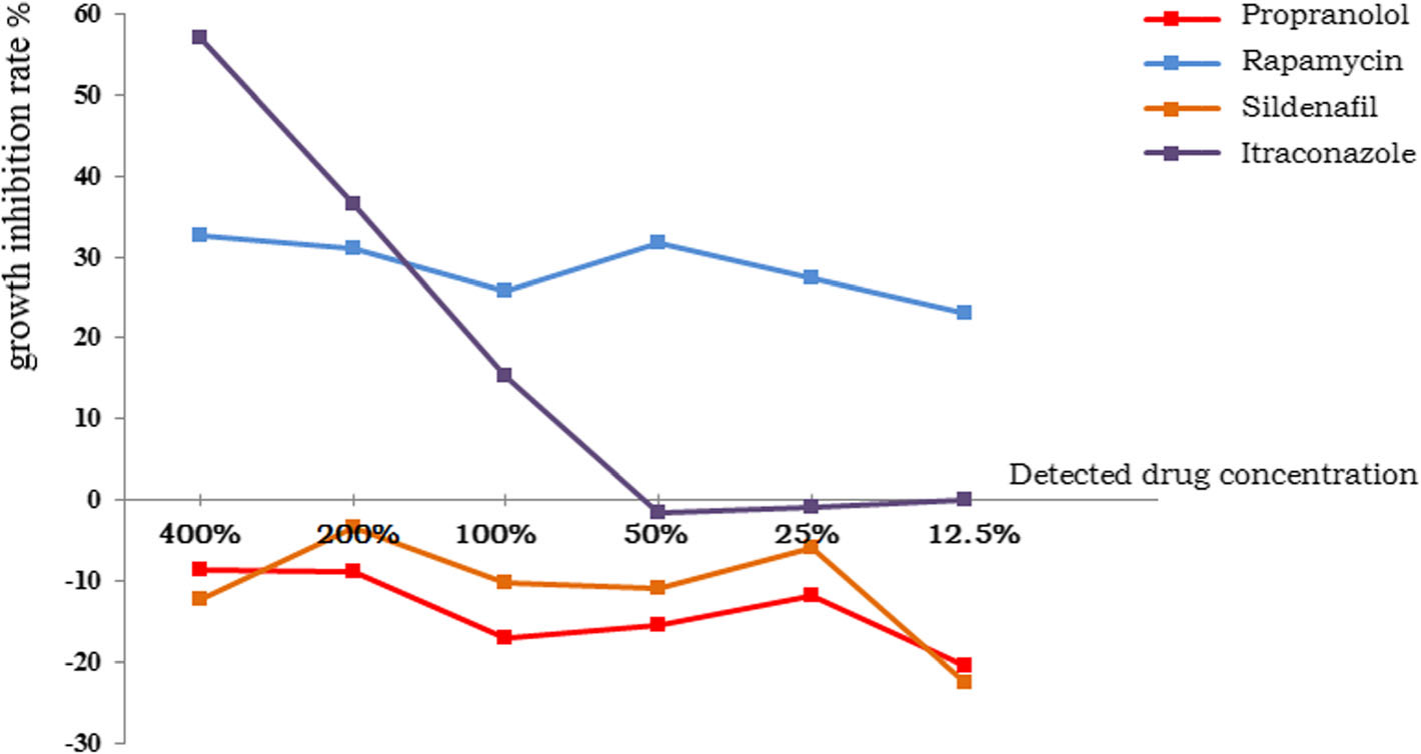
The average inhibition curves of capillary malformations group

During the incubated days, the growths of vascular anomalies specimens’ cells were observed daily, to make sure that cells populations were inoculated distributed evenly without aggregation and no bacteria, fungi or mycoplasma contamination was found. Three days later, adenosine triphosphates were extracted from the suspensions and the fluorescence intensity of these specimens at 560 nm wavelength was measured. Then, the actual inhibition rates were calculated and determined (Table 1, Table 2). The average inhibition rates of each drug in the two groups were calculated respectively (Table 3, Table 4), and the inhibition curves were drawn (Fig. 1, Fig. 2).

**Table 1:** Testing result of 5 cases infantile hemangiomas specimens, growth inhibition rates (%)

Testing drug	400%	200%	100%	50%	25%	12.5%	TGI
Patient No.1							
Propranolol	18.04	8.83	9.95	5.34	3.81	1.16	47.13
Rapamycin	19.02	44.87	19.10	25.21	16.75	24.26	149.21
Sildenafil	2.13	−9.31	−14.08	−19.25	−38.13	−46.28	− 124.92
Itraconazole	49.77	58.43	18.98	−22.85	8.56	17.01	129.9
Patient No.2							
Propranolol	27.99	18.03	22.05	18.63	18.59	20.94	126.23
Rapamycin	27.06	14.24	5.91	5.28	87.19	10.31	149.99
Sildenafil	15.02	−29.00	−13.13	−23.39	−12.60	−8.77	−71.87
Itraconazole	12.06	45.79	53.04	25.30	−8.01	53.94	182.12
Patient No.3							
Propranolol	23.06	23.27	27.94	0.86	11.55	32.94	119.62
Rapamycin	42.99	44.02	35.61	38.37	39.46	32.60	233.05
Sildenafil	−11.26	−1.60	−13.39	− 10.91	−1.42	− 15.48	− 54.06
Itraconazole	32.02	23.12	13.06	2.13	−8.28	−10.31	51.74
Patient No.4							
Propranolol	32.76	13.54	40.21	36.66	42.37	31.43	196.97
Rapamycin	13.03	56.73	8.15	50.81	45.72	11.57	186.01
Sildenafil	−6.90	16.45	18.61	26.69	15.26	−7.27	62.84
Patient No.5							
Propranolol	4.29	6.24	0.50	11.70	8.31	13.53	44.57
Rapamycin	42.84	56.01	52.12	48.74	47.45	37.93	285.09
Sildenafil	12.36	8.49	−7.87	5.00	−7.87	−7.90	2.21

*TGI* total growth inhibition rate, summation of the percentage of growth inhibition at every testing concentration

**Table 2:** Testing result of 11 cases capillary malformations specimens, growth inhibition rates (%)

Testing drug	400%	200%	100%	50%	25%	12.5%	TGI
Patient No.1							
Propranolol	−6.98	−19.18	−14.02	−15.42	−7.98	−13.91	−77.49
Rapamycin	10.71	−15.56	−8.48	19.28	12.73	10.09	28.77
Sildenafil	−18.21	−3.58	2.79	−19.52	−12.89	− 8.21	−59.62
Itraconazole	61.01	35.15	10.15	−2.34	−11.28	−3.06	89.63
Patient No.2							
Propranolol	−23.96	7.19	−6.83	−25.35	3.14	−16.09	− 61.90
Rapamycin	30.14	29.28	7.05	13.38	24.11	−6.67	97.29
Sildenafil	−24.71	−10.00	− 25.87	−4.61	−12.19	− 47.52	− 124.9
Itraconazole	58.00	20.68	6.96	−17.90	− 18.74	−9.75	39.25
Patient No.3							
Propranolol	−43.29	−19.52	−15.63	−3.46	−8.10	− 19.72	− 109.72
Rapamycin	28.18	48.20	49.61	59.12	45.48	42.99	273.58
Sildenafil	−3.51	8.34	−7.31	−8.34	−10.17	−14.19	− 35.18
Itraconazole	27.82	29.63	−3.77	−8.82	−9.20	−8.41	27.25
Patient No.4							
Propranolol	−14.52	−6.70	11.50	9.24	−15.89	−8.00	−24.37
Rapamycin	61.07	61.52	59.12	62.74	58.41	60.01	362.87
Sildenafil	−13.62	−12.51	−15.82	− 19.69	−20.55	−22.43	− 104.62
Itraconazole	48.74	17.26	−3.79	−13.68	−9.71	−6.65	32.17
Patient No.5							
Propranolol	−25.19	− 24.47	− 15.96	−5.67	−9.79	−14.35	− 95.43
Rapamycin	33.80	37.30	36.81	36.86	37.58	37.39	219.74
Sildenafil	−9.62	5.53	9.88	5.10	5.56	9.34	25.79
Itraconazole	55.08	52.15	34.02	12.58	22.94	14.98	191.75
Patient No.6							
Propranolol	25.94	6.19	−10.22	−26.36	−18.57	−50.00	−73.02
Rapamycin	13.42	−11.34	−41.93	0.35	−19.40	−16.90	−75.8
Sildenafil	−1.60	17.59	−5.49	−3.55	38.46	3.42	48.83
Itraconazole	91.59	57.65	46.24	−2.16	7.02	−0.21	200.13
Patient No.7							
Propranolol	19.12	10.04	13.45	−12.48	6.71	9.44	46.28
Rapamycin	28.33	28.26	27.07	19.75	17.94	16.43	137.78
Sildenafil	12.78	17.26	−11.37	3.84	−0.74	−21.08	0.69
Itraconazole	57.51	42.83	16.52	21.09	13.09	12.72	163.76
Patient No.8							
Propranolol	−3.20	−1.04	−10.86	−17.05	− 14.17	0.83	−45.49
Rapamycin	2.01	5.93	−1.23	−6.97	−17.91	−5.67	−23.84
Sildenafil	4.26	−6.58	−10.47	−17.54	−25.50	−6.80	−62.63
Patient No.9							
Propranolol	−27.68	−29.55	−42.66	−33.58	− 29.81	−73.67	− 236.95
Rapamycin	28.00	26.94	28.25	20.65	31.59	−3.94	131.49
Sildenafil	−58.38	−57.51	−47.19	− 49.11	−46.99	− 118.09	− 377.27
Patient No.10							
Propranolol	7.06	−3.30	−10.75	−16.81	− 10.72	15.52	− 19
Rapamycin	47.64	51.80	51.03	47.49	39.85	44.70	282.51
Sildenafil	17.91	2.59	5.02	−1.91	−0.90	16.28	38.99
Patient No.11							
Propranolol	−1.52	− 18.04	−86.25	−23.50	−24.81	−56.10	− 210.22
Rapamycin	74.36	79.44	75.57	75.02	70.64	73.95	448.98
Sildenafil	−39.53	1.07	−7.27	−4.22	20.95	− 38.48	−67.48

**Table 3:** The average growth inhibition rates (%) of infantile hemangiomas group

		400%	200%	100%	50%	25%	12.5%	TGI
Propranolol	*n* = 5	21.228	13.982	20.130	14.638	16.926	20.000	106.904
Rapamycin	*n* = 5	28.988	43.174	24.178	33.682	47.314	23.334	200.670
Sildenafil	*n* = 5	2.270	−2.994	−5.972	−4.372	− 8.952	−17.14	−37.160
Itraconazole	*n* = 3	31.283	42.446	28.36	1.526	−2.576	20.213	121.253

*TGI* total growth inhibition rate, summation of the percentage of growth inhibition at every testing concentration

**Table 4:** The average growth inhibition rates (%) of capillary malformations group

		400%	200%	100%	50%	25%	12.5%	TGI
Propranolol	*n* = 11	−8.565	−8.943	−17.111	− 15.494	−11.817	−20.550	− 82.482
Rapamycin	*n* = 11	32.514	31.071	25.715	31.606	27.365	22.943	171.215
Sildenafil	*n* = 11	−12.202	−3.436	−10.281	− 10.868	−5.905	−22.523	−65.218
Itraconazole	*n* = 7	57.107	36.478	15.192	−1.604	−0.840	−0.0542	106.277

*TGI* total growth inhibition rate, summation of the percentage of growth inhibition at every testing concentration

In infantile hemangiomas group (Table 1), no matter in individual patient or seen as a whole, propranolol showed inhibitory effects on infantile hemangiomas cells evenly. The growth inhibition rate of propranolol in some cases was even higher than (patient No.4) or close to (patient No. 2) that of rapamycin. Rapamycin and itraconazole also showed inhibitory effects on infantile hemangiomas cells. As the number of cases is too small (*n* = 3), the data of itraconazole fluctuated greatly. Except for some isolated data of patient No. 4, the testing result of sildenafil is mostly negative numbers. Just be explained from the experimental results, far from inhibiting the growth of infantile hemangiomas cells, sildenafil even promotes the growth of them on the contrary. Therefore, it can be considered that propranolol, rapamycin and itraconazole can inhibit the growth of infantile hemangiomas cells with different rates, and among them, rapamycin is the most effective. Sildenafil has no growth inhibitory effect on infantile hemangiomas cells.

In capillary malformations group (Table 2), the testing results of propranolol were significantly different from which in infantile hemangiomas group. Apart from several individual data, propranolol generally had even no inhibitory effect or little inhibitory effect (patient No.7) on capillary malformation cells. Sildenafil have appeared similar testing results, and also had no inhibitory effect on capillary malformation cells except several individual data. Both rapamycin and itraconazole indicated inhibitory effects on capillary malformation cells. Remarkably and noteworthily, with the increase of its concentration, itraconazole showed a significant trend that the inhibition rates were increasing dramatically to some extent, but while the concentration decreased, its inhibitory effect on capillary malformation cells were lost. In general, rapamycin can inhibit the growth of capillary malformations cells; itraconazole can inhibit the growth of capillary malformations cells only within high concentrations; propranolol and sildenafil has no growth inhibitory effect on capillary malformations cells.

## Discussion

The idea of introducing and carrying out personalized drug sensitivity test in vitro is not new, and researchers have been attempting to develop much higher precision in vitro methods to predict therapeutic response for various diseases such as tumors [22] or infectious illnesses [23] for several decades. Among these techniques and methods, adenosine triphosphate-tumor chemosensitivity assay (ATP-TCA) [24] is chosen as the testing method in this study for its convenient and high sensitivity. ATP-TCA compares the intracellular ATP levels of drug-exposed cells and untreated controls (M0) to evaluate the growth inhibition effect, and could accurately predict the therapeutic response of different drugs in certain cancer. It does not need any special complicated instruments or equipments, and the number of tissues cells needed to be detected is few, which can be as low as 50–250. It has no dependence on cell proliferation situation, and can also detect the inhibition effect of drugs on G0 phase cells. Beyond tumor or entities cells themselves, some therapeutics drugs could also target the associated extracellular matrix or microenvironment, and ATP-TCA can detect the overall growth inhibitory effect of certain drugs on tumor tissues cells, with the advantages of needing not to isolate, purify or cultivate tumor cells specifically and deliberately. Thus, this testing method is more suitable for those infant patients who were hardly to excise enough tissues, and contributes to obtain the overall evaluation of drug efficacy.

According to the testing result, in infantile hemangiomas group, though rapamycin or itraconazole appeared to be more effective in inhibiting the growth of hemangiomas cells than propranolol, it is not recommended to prescribe rapamycin or itraconazole in clinical for the consideration of their widely adverse reactions and the anxiety from parents. Infantile hemangiomas are a special type of benign tumour with a distinctive characteristic that can regress or subside spontaneously during their natural history. Therefore, the choices of therapy approaches are required to find a balance between the effect of antitumor and the efforts to reduce side or adverse reactions. Those over-zealous attempts that to perform aggressively treatments only to pursue a high inhibition rate partially should be avoided [25]. On the other hand, for those infants who were contraindicated to propranolol (contraindication), failed to respond to propranolol (refractory), or hemangiomas regrown after propranolol treatment (recurrence), rapamycin or itraconazole may serve as a choice after comprehensive evaluation.

In capillary malformations group, rapamycin showed a relative smoother inhibition curve, while itraconazole showed a dose-dependent inhibition curve to some extent. Itraconazole is known as a highly efficacious broad spectrum antifungal agent for many years, however, a series of research recently have found that it also has some unknown effects on antineoplastic and immunoregulation by some mechanism such as inhibiting endothelial cells and angiogenesis [26]. This suggests that itraconazole can be seen as a treatment for angiogenesis-dependent diseases. Nevertheless, what needed to be concerned is that itraconazole acts in a dose-dependent manner and requires a higher concentration to perform its inhibition effect, and at low level of concentration its inhibition rate is gradually declined and significantly less than that of rapamycin. Obviously, when in vivo, it is difficult to maintain high blood concentration all the time due to the influence of metabolic related factors. Therefore, compared with itraconazole, rapamycin is more inclined to recommend for children with vascular malformations.

Rapamycin has exhibited similar inhibition effect both in infantile hemangiomas group and in capillary malformations group, while propranolol shows very different manifestations. Propranolol is ineffective for capillary malformations cells, and at present there is no news or report available about the success of using propranolol in treating any vascular malformation [27]. This indicates that rapamycin may have broad-spectrum inhibitory effect on angiogenesis-relevant diseases, while propranolol only relatively specifically target hemangiomas cells through some special mechanisms [28]. These different pharmacological mechanisms of rapamycin and propranolol may provide new ideas for further study of vascular anomalies.

It is worth noting that sildenafil did not show any inhibitory effect in either infantile hemangiomas group or capillary malformations group at least in this study. Although sildenafil has been reported to be effective in multiple vascular anomalies, diverse opinions persisted all through. Sildenafil maybe caused a significant amelioration in the responsiveness or endothelial function of various vascular, but caution is recommended prior to use in clinical [29].

Through ATP-TCA, which drug has a higher inhibition rate on infant subject’s anomalies tissues is much more clear. However, especially in the pediatrics clinical, therapeutic approaches or prescription drugs cannot be selected doctrinaire and avoiding making choices directly completely according to the experimental results without any analysis. It is necessary to evaluate the specific conditions of each infant, combined with the adverse reactions and metabolism pattern of each drug, and then, to give individualized medication suggestions to meet the particular individual needs.

Furthermore, more high-quality trials with a large sample and longer follow-up are required, and continuation in-depth study will be carried out both in laboratory and clinical practice: to grope the proper and more suitable conditions for the culture of vascular anomalies cells; to improve, reform and optimize the experimental procedures; to compare the inhibition effect of more drugs such as atenolol, timolol etc., and combine with clinical efficacy and follow-up results; to carry out multi-factor analysis; and so on. After summarizing and concluding these data, to find some general regularity and to provide a basis for the standardized treatment of vascular anomalies in the future.

## Conclusions

Adenosine triphosphate sensitivity assay is a sensitive assay and suitable for use in clinical settings. Through this experiment, it is clear which drug has a strong inhibition rate on infant subject’s anomalies tissues, thus individualized medication suggestions were offered together with a comprehensive evaluation of each infant before the management of vascular anomalies. Moreover, this assay also has notable advantages for guiding the design of therapy protocols and assessing novel therapeutic drugs.

## Data Availability

Not applicable.

## Abbreviations

ATP-TCA: Adenosine triphosphate-tumor chemosensitivity assay
AUC: Area under curve
IC50: 50% inhibition rate
IC90: 90% inhibition rate
ISSVA: The International Society for the Study of Vascular Anomalies
M0: Drug-free control, only culture medium and lesions tissues, without any drug
MI: Maximum inhibitory control, only culture medium
SI: Sensitivity index
TGI: Total growth inhibition rate
